# Identification of pathogenic variants for the development of ultra-long axial length in myopic children

**DOI:** 10.1371/journal.pone.0337184

**Published:** 2025-11-19

**Authors:** YanYing Zhu, XueYan Li, YueXin Chen, HaiYan Xie, YuKun Liu, XiaoChen Xu, Jing Wang

**Affiliations:** 1 Department of Ophthalmology, The Second Affiliated Hospital of Anhui Medical University, Hefei, Anhui Province, China; 2 Department of Ophthalmology, Wuhu City Eye Hospital, Wuhu, Anhui Province, China; Aravind Eye Hospital, INDIA

## Abstract

**Purpose:**

Axial elongation is a key factor in myopia progression, yet its genetic basis remains incompletely understood. This study aims to identify pathogenic genetic variants associated with excessively elongated axial length in children.

**Methods:**

This study included 56 children with axial lengths exceeding the normal range for their age group, and whole-exome sequencing (WES) was performed on their oral mucosal samples. Clinical evaluations included axial length measurement, refraction testing, and fundus photography to assess the degree of myopia and retinal changes. Co-segregation analysis was conducted in selected families (F#1, F#2, F#5) to validate the familial inheritance patterns of the variants.

**Results:**

Fifteen children carried variants in genes including BBS2, OPN1LW, P4HA2, FBN1, LOXL3, FZD4, USH2A, COL2A1, and BFSP2, with five novel variants identified: BBS2 (c.700C > T), P4HA2 (c.1382C > G), FBN1 (c.7130T > C), LOXL3 (c.1580delC), and FZD4 (c.1315G > A). Notably, a rare compound heterozygous BBS2 variant (c.700C > T/c.534 + 1G > T) was found in a non-syndromic child, and the P4HA2 (c.419A > G) variant in family F#5 exhibited a phenotype distinct from previous studies.

**Conclusions:**

This study identified five novel variants sites and discovered two cases with phenotypes distinct from previous studies, thereby expanding the genetic variant spectrum associated with myopia and providing new targets for genetic screening and intervention.

## Introduction

Myopia is a global public health concern, with its prevalence increasing annually worldwide, accompanied by trends of earlier onset and greater severity [1]. In China, the incidence of myopia among children aged 6–8 years increased from 10–15% before the COVID-19 pandemic to 20–25% during the pandemic, and stabilized at 15–18% post-2021 [2]. The elongation of the axial length, driven by genetic and environmental factors, is the primary cause of myopia progression, although its genetic mechanisms remain not fully understood. As the axial length gradually increases in children and adolescents, the risk of developing high myopia and retinal complications also rises, which severely affects vision health and can even lead to blindness [3]. Therefore, monitoring changes in axial length is critical for predicting myopia progression.

Genetic factors play a significant role in the development of myopia. Research suggests that the genetic mechanisms contributing to axial elongation may involve multiple biological pathways, including scleral remodeling and collagen metabolism abnormalities (e.g., P4HA2 gene variants associated with non-syndromic high myopia [4]); genetic defects in connective tissue structure and elasticity (e.g., FBN1 variants linked to Marfan syndrome [5]); dysregulation of retinal signaling (e.g., OPN1LW and OPN1MW genes related to retinal function [6]); developmental anomalies of the lens or vitreous (e.g., BFSP2 and COL2A1 variants [7,8]); and other genes associated with genetic syndromes [9,10]. These mechanisms, through genetic variants, directly or indirectly affect the structure and growth of the eyeball, potentially leading to abnormal axial length elongation.

Studies have indicated that axial length and refractive error are regulated by shared genetic factors [11–13]. Currently, genes associated with axial length listed in the Online Mendelian Inheritance in Man (OMIM) database include CPSF1 [14], ADAMTSL4 [15], SOX2 [16], MCOP6 [17], and others. However, these genes can only account for a subset of cases related to axial elongation, while the mechanisms linking most genes to phenotypes remain unresolved, and the complete genetic profile remains incomplete. This is particularly evident in pediatric populations, where early-onset myopia may reflect a stronger genetic influence.

This study employed WES to explore novel genetic variants associated with excessively elongated axial length in children, aiming to deepen the understanding of myopia progression and provide a basis for intervention.

## Methods

### Patients

This study was approved by the Ethics Committee of the Second Affiliated Hospital of Anhui Medical University (approval No. SL-YX2023–092). Written informed consent was obtained from the legal guardians of all participants, and oropharyngeal swab samples were collected for genetic analysis with their consent. The Ethics Committee granted a waiver of separate informed consent for the collection of oropharyngeal swab samples. All samples and data were coded and de-identified during the study, ensuring that researchers could not directly link them to specific individuals, thereby safeguarding participant privacy. The study adhered to the ethical principles outlined in the Declaration of Helsinki.

A total of 56 children aged 6–12 years who attended the ophthalmology outpatient clinic of our hospital between 19 October 2023 and 18 October 2024 were enrolled in this study. These children had axial lengths exceeding the normal range for their age group, based on data published by the Ophthalmology Branch of the Chinese Preventive Medicine Association in 2022 [18]. Children with other ocular diseases, a history of ocular surgery, or syndromic conditions were excluded. Clinical assessments included axial length measurement using the IOLMaster 700 (Carl Zeiss Meditec, Germany), refraction testing, and fundus photography to evaluate the degree of myopia and retinal changes.

### Experimental methods

Oral mucosal cells were collected using oral swabs from the patients, and genomic DNA was extracted using the MagMax™ DNA multi-Sample Ultra 2.0 Kit (Thermo Fisher Scientific), according to the manufacturer’s instructions. The WES technology utilized the Twist Human Core Exome Kit and the NovaSeq 6000 platform (Illumina, San Diego, USA) was employed to obtain genetic information. A paired-end sequencing strategy (sequencing from both ends of the fragments) was used to generate high-quality sequencing data (reads), with a target sequencing depth of 100x and an average read length of 150 bp.

The raw sequencing data in FASTQ format were subjected to quality control using FastQC and Trimmomatic. The cleaned data were then aligned to the GRCh37 reference genome using BWA-MEM [19]. Variant calling was performed using GATK v4.2 [20], including variant detection with HaplotypeCaller. Variants were annotated using ANNOVAR [21], with the prediction tools of Combined Annotation-Dependent Depletion (CADD v1.7) [22], REVEL [23], SpliceAI [24], SIFT [25], MutationTaster2021 [26], PolyPhen-2 [27] and AlphaMissense [28] as well as the gnomAD [29] and ClinVar [30] databases. The following filtering criteria were applied: (1) AF < 0.005 in GnomAD4_exome WBBC、ChinaMAP database; (2) CADD score > 20.

Based on the guidelines published by the American College of Medical Genetics and Genomics (ACMG) and the application recommendations from the ClinGen Sequence Variant Interpretation (SVI) expert group, clinical interpretation aims to provide a systematic evaluation of genetic variants [31]. This assessment process incorporates pathogenic evidence (PVS1, PM1-PM6, PP1-PP5) and benign evidence (BS1-BS4, BA1) to classify variants. According to the ACMG guidelines, variants are classified into the following categories: “Pathogenic,” “Likely Pathogenic,” “Variant of Uncertain Significance (VUS),” “Likely Benign,” and “Benign.” This classification approach provides a comprehensive evaluation of genetic variants, helping to determine their potential role in disease development.

## Results

Among the 56 children, the median age was 9 years. The clinical characteristics of the 15 children carrying genetic variants are summarized in Table 1. All tested individuals were free of other ocular diseases that could affect refractive status (e.g., glaucoma, cataracts, corneal diseases), had no history of ocular surgery, and did not have syndromic conditions. Segregation analysis was performed for families F#1, F#2, and F#5 in this study.

**Table 1:** Clinical features of 15 variant-carrying children.

Family (Proband)	sex	age	SE (DS)		AL(mm)	
			OD	OS	OD	OS
F#1	F	4	−7	−7	25.32	25.31
F#2	F	10	−7.125	−7.625	25.13	25.2
F#3	M	3	−6.125	−6	25.66	25.54
F#4	M	3	−6	−5	25.24	24.81
F#5	M	6	−1.75	−1.75	24.64	24.6
F#6	M	8	−5.75	−6	25.26	25.22
F#7	M	8	−5.65	−6.125	25.3	25.14
F#8	M	11	−3.5	−3.25	26.99	26.43
F#9	M	3	−6.75	−7.5	24.14	24.51
F#10	M	9	−2.5	−2.75	25.22	25.19
F#11	M	12	−4.25	−4.25	27.05	27.07
F#12	M	9	−2.25	−2.1	25.44	25.28
F#13	F	7	−4.25	−4.25	24.51	24.62
F#14	M	8	−4	−2.25	25.91	25.28
F#15	F	7	−3.75	−3	24.42	24.74

F,female;M,male;OD, right eye; OS, left eye; AL, axial length;SE Spherical equivalent.

Among the 56 patients, 15 cases with suspected variants were identified(Table 2), involving variants in nine genes: BBS2, OPN1LW, P4HA2, FBN1, LOXL3, FZD4, USH2A, COL2A1, and BFSP2. These included five novel potentially pathogenic variants not previously reported: BBS2 (c.700C > T), P4HA2 (c.1382C > G), FBN1 (c.7130T > C), LOXL3 (c.1580delC), and FZD4 (c.1315G > A).

**Table 2:** Clinical characteristics and genetic variants carried by 15 patients.

Family (Proband)	Gene	Transcript	DNA Change	Predicted amino acid change	Variant Type	Inheritance	ACMG Classification	SIFT/MutTaster/PolyPhen	CADD_Phred	REVEL	SpliceAI	AlphaMissense	GnomAD4_exome	WBBC/ChinaMAP	HGMD	PubMed
F#1	BBS2	NM_001377456.1	c.700C > T	p.Arg234*	nonsense variant	AR	P (PVS1 + PM3 + PM2)	-/-/-	38	–	0.04	–	1.51E-05	1.1E-4/3.9E-4	DM	novel
	BBS2	NM_001377456.1	c.534 + 1G > T	–	splice donor	AR	P (PVS1 + PP1 + PM3 + PM2)	-/-/-	32	–	0.99	–	1.10E-05	2.2E-4/4.4E-4	–	PMID:33777945,33520300,24280758
F#2	OPN1LW	–	–	LVAVA(exon3)	–	–	–	–	–	–	–	–	–	–	–	PMID: 34440353,26114493,37097228,25168334
F#3	OPN1LW	–	–	LVAVA(exon3)	–	–	–	–	–	–	–	–	–	–	–	PMID: 34440353,26114493,37097228,25168334
F#4	OPN1LW	–	–	LVAVA(exon3)	–	–	–	–	–	–	–	–	–	–	–	PMID: 34440353,26114493,37097228,25168334
F#5	P4HA2	NM_001017974.2	c.419A > G	p.Gln140Arg	missense variant	AD	VUS (PM2 + PP3)	D/D/D	25.7	0.93	0	0.9042	9.58E-06	1.3E-3/6.4E-4	DM	PMID: 25741866
F#6	P4HA2	NM_001017974.2	c.871G > A	p.Glu291Lys	missense variant	AD	LP (PP1 + PM2 + PP3)	D/D/D	29.6	0.82	0.01	0.8696	1.16E-05	6.7E-4/3.9E-4	DM	PMID: 25741866
F#7	P4HA2	NM_001017974.2	c.1382C > G	p.Ala461Gly	missense variant	AD	VUS (PM1 + PM2 + PP3)	D/D/D	30	0.51	0	–	3.42E-06	3.3E-4/2.0E-4	–	novel
F#8	FBN1	NM_000138.5	c.7130T > C	p.Ile2377Thr	missense variant	AD	VUS (PM2,PP3)	D/D/D	24.1	0.67	0	0.3454	6.84E-07	0/0	–	novel
F#9	FBN1	NM_000138.5	c.4261C > T	p.Leu1421Phe	missense variant	AD	VUS (PM1 + PM2 + PP2 + PP3)	D/D/D	26.8	0.88	0	0.3284	6.84E-07	0/0	DM	PMID: 11059536
F#10	LOXL3	NM_032603.5	c.1580delC	p.Thr527fs	frameshift	AR	LP (PVS1 + PM2)	-/-/-	–	–	–	–	0	0/0	–	novel
F#11	FZD4	NM_012193.4	c.1315G > A	p.Val439Met	missense variant	AD	VUS (PM1 + PM2 + PP3)	D/D/D	25.9	0.72	0	–	0	0/0	–	novel
F#12	USH2A	NM_206933.4	c.4616C > T	p.Thr1539Ile	missense variant	AR	VUS (PM1 + PM2 + PS1 + PP3)	D/D/D	20.8	0.23	0.05	0.2489	2.60E-05	1.3E-3/1/3E-3	–	PMID: 23967202
	USH2A	NM_206933.4	c.2209C > T	p.Arg737*	stop gained	AR	P (PVS1 + PM1 + PM2)	-/-/-	36	–	0.19	–	4.79E-06	0/0	DM	PMID: 17296898
F#13	COL2A1	NM_001844.5	c.3106C > T	p.Arg1036*	stop gained	AD	P (PVS1 + PM2 + PP5)	-/-/-	45	–	0.02	–	6.84E-07	0/0	DM	PMID: 16752401,20179744,20513134
F#14	BFSP2	NM_003571.4	c.1016G > A	p.Arg339His	missense variant	AD	LP (PM2 + PP1 + PP3)	D/D/D	22.7	0.671	0.01	0.0981	1.10E-05	0/0	DM	PMID: 18958306
F#15	BFSP2	NM_003571.4	c.859C > T	p.Arg287Trp	stop gained	AD	P (PVS1 + PP3)	D/D/D	26.3	0.737	0	0.1363	2.33E-05	0/0	DM	PMID: 10729115

F,Family; P, pathogenic; LP, likely pathogenic; VUS, variants of unknown significance.

Among the variant types, missense variants were the most prevalent, accounting for 7 cases, potentially influencing scleral changes associated with myopia or accelerating disease progression by altering protein structure or function. This was followed by nonsense variants (2 cases), frameshift variants (1 case), complex missense variants (1 case), and a combined nonsense and splice site variant (1 case). This distribution suggests that multiple mechanisms can lead to impaired gene function, with amino acid substitution variants (missense) being the predominant type, consistent with the variant spectrum characteristics observed in previous genetic studies of myopia and ocular development.

**Fig 1:**
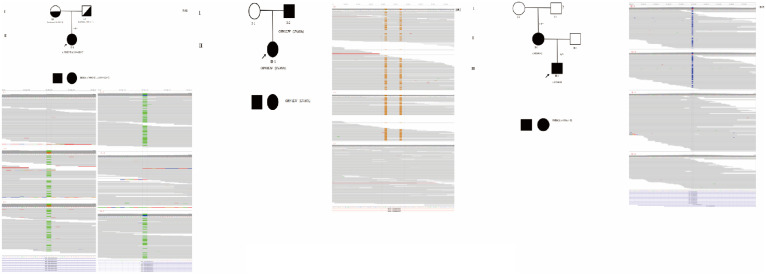
Pedigrees and IGV diagrams for F#1, F#2, and F#5. The pedigree and IGV diagrams for F#1, F#2, and F#5 depict variant status. Squares represent males, circles represent females; filled (black) and unfilled (white) symbols indicate affected and unaffected individuals, respectively, while half-filled symbols denote unaffected carriers. The proband is indicated by an arrow. The genotype + /- signifies heterozygous individuals.

WES and a series of ophthalmic examinations, including visual acuity, axial length, refractive error, fundus photography, retinal nerve fiber layer thickness, visual evoked potentials (VEP), and macular optical coherence tomography (OCT), were performed on two generations of the F#1 patient’s family (Fig 1). The proband was identified with a novel compound heterozygous variant in the BBS2 gene (c.700C > T/c.534 + 1G > T). The c.700C > T variant, a newly discovered nonsense variant, was inherited from the father, while the c.534 + 1G > T variant was inherited from the mother. Both parents carry one mutated allele without exhibiting symptoms, confirming the recessive inheritance pattern [10]. The novel variant c.700C > T (p.Arg234*) is a nonsense variant located in the coding region of the BBS2 gene, resulting in premature protein termination and predicted to be a loss-of-function variant.

**Fig 2:**
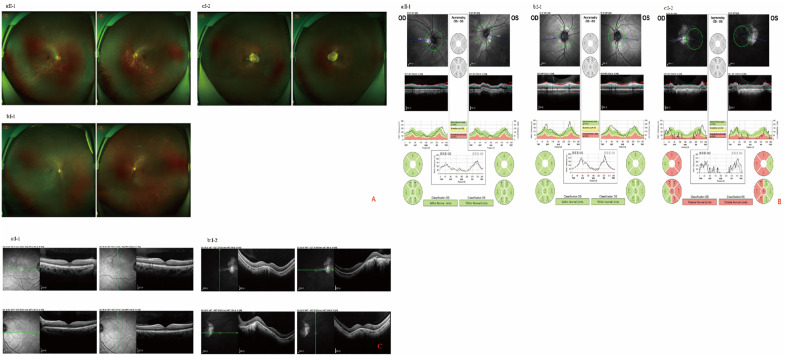
Wide-angle fundus images and RNFL thickness analysis for F#2 pedigree. A represents wide-angle fundus photographs of the F#2 pedigree: (a) fundus displays leopard-spot changes, (b) fundus shows no abnormalities, (c) fundus exhibits leopard-spot changes and diffuse retinal atrophy. B represents retinal nerve fiber layer (RNFL) thickness analysis of the F#2 pedigree: (a, b) no abnormalities, (c) indicates thinning of the RNFL. C represents the OCT examination in F#2, where the retinal structure in the macular region appears essentially normal in (a, b).

WES and a series of ophthalmic examinations (including visual acuity, axial length, refractive error, fundus photography, retinal nerve fiber layer thickness, VEP and OCT) were conducted on two generations of the F#2 family (Fig 2). Both the father and the affected child were found to have the OPN1LW (LVAVA) variant, both exhibiting elongated axial lengths and high myopia. Fundus examination revealed a leopard spot-like pattern of retinal changes, with the father showing optic disc atrophy and thinning of the retinal nerve fiber layer. This finding is consistent with the study by Neitz M et al [6].

Guo et al. (2015) [4] confirmed that P4HA2 c.419A > G (p.Q140R) is associated with non-syndromic high myopia. In this study, the patient (F#5) is a 6-year-old child, with an axial length of 24.64 mm in the right eye and 24.60 mm in the left eye, both exceeding the normal range for children of the same age (20.93 mm–23.98 mm [18]), though the deviation is not statistically significant. The patient exhibited mild myopia (right eye SE: −1.75D, left eye SE: −1.75D), consistent with the observed axial length.

**Fig 3:**
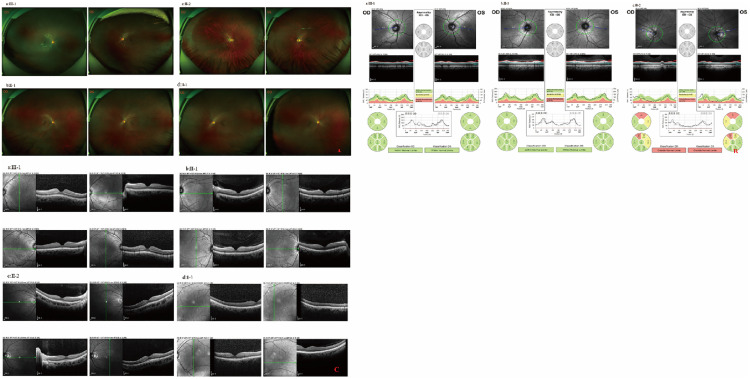
Wide-angle fundus images and RNFL thickness analysis for F#5 pedigree. A represents wide-angle fundus photographs of the F#5 pedigree: (b, c) fundus displays leopard-spot changes, (a, d) fundus shows no abnormalities. B represents RNFL thickness analysis of the F#5 pedigree: (a, b) no abnormalities, (c) indicates thinning of the RNFL.C represents the OCT examination in F#5, where the retinal structure in the macular region appears essentially normal in (a, b, c, d).

Subsequently, WES and a series of ophthalmic examinations (including visual acuity, axial length, refractive error, fundus photography, retinal nerve fiber layer thickness, VEP, and OCT) were performed on three generations of the family of the patient. (Fig 3) The patient’s mother, 35 years old, also carried the heterozygous variant and exhibited moderate myopia (right eye SE: −3.50D, left eye SE: −3.75D), with bilateral axial lengths of 24.59 mm, consistent with her refractive status. The patient’s father, 38 years old, presented with pathological high myopia, but no abnormal variants were detected. His right eye spherical equivalent refractive error was −7.375D, and the left eye was −6.25D, with right and left axial lengths of 27.71 mm and 27.33 mm, respectively. Fundus examination revealed leopard spot-like changes. No other family members carried the variant.

The P4HA2 c.1382C > G (p.A461G) variant in F#7 has not been reported in PubMed, Google Scholar, ClinVar, or gnomAD databases (accessed March 2025). This is a missense variant located in the coding region of the P4HA2 gene, resulting in the substitution of alanine with glycine.

A novel variant site, FBN1 (c.7130T > C), was detected in F#8. This specific variant has not been clearly associated with Marfan syndrome (MFS) in major genetic variation databases such as ClinVar and HGMD, and the patient exhibited significant axial elongation and refractive error. This is a missense variant located in the coding region of the FBN1 gene, resulting in the substitution of isoleucine with threonine.

A novel variant, c.1580delC (p.Thr527fs), in the LOXL3 gene was identified in F#10. This variant is a frameshift variant caused by a single nucleotide deletion, located in the coding region of the LOXL3 gene, leading to a shift in the translation frame and predicting the production of a truncated protein.

A novel variant, c.1315G > A (p.Val439Met), in the FZD4 gene was identified in F#11. This is a missense variant located in the coding region of the FZD4 gene, resulting in the substitution of valine with methionine.

## Discussion

Myopia is a focal point of global ophthalmic research, with axial elongation being the core pathological feature of myopia. Genetic factors play a critical role in its development. Understanding the role of genes in the axial length of children and adolescents can help prevent high myopia and its complications, and provide insights for the development of future axial management strategies.

Variants in the BBS2 gene are closely associated with severe visual impairment in patients with Bardet-Biedl syndrome (BBS) [10]. This gene encodes a ciliary protein primarily implicated in visual dysfunctions such as retinitis pigmentosa (RP), rather than axial elongation. This study identified a rare case of a non-syndromic child carrying a compound heterozygous BBS2 variant (c.700C > T/c.534 + 1G > T), with the only manifestations being myopia and axial elongation, which differs from the report by Meng et al. (2021) [10]. These findings suggest a potential novel role of BBS2 in myopia, or possibly an early pre-symptomatic stage of BBS, warranting further investigation.

The FZD4 (Frizzled-4) gene encodes a receptor in the Wnt signaling pathway, which is critically involved in retinal vascular development. Previous studies have demonstrated that the Norrin/FZD4 signaling axis activates the canonical Wnt/β-catenin pathway, regulating the proliferation and migration of retinal endothelial cells to maintain angiogenesis [32]. In subject F#11, a novel FZD4 variant (c.1315G > A) was detected, accompanied by axial elongation (axial length: 27.05 mm/27.07 mm). This variant may reduce Norrin signaling, potentially leading to impaired retinal vascular development, which could subsequently influence scleral remodeling and contribute to axial elongation. These findings suggest a potential role for retinal-related pathways in axial growth.

A novel variant in P4HA2, c.1382C > G (p.A461G), has been identified, which, as of March 2025, has not been reported in PubMed, Google Scholar, ClinVar, or gnomAD databases. This variant is located within the dioxygenase domain (approximately residues 200–500 of the gene) and is in close proximity to previously known variants, c.1327A > G (p.K443*) and c.1349_1350delGT (p.R451Glyfs*8) [4], with a distance of approximately 10–18 amino acids between them. This clustering underscores the critical functional role of this region in enzyme activity. Given the severe loss-of-function effects observed in the neighboring variants, the c.1382C > G variant may similarly impair collagen hydroxylation, potentially reducing scleral tensile strength and promoting axial elongation.

Previous studies have indicated that LOXL3 encodes lysyl oxidase-like protein 3, which is responsible for the crosslinking of collagen and elastin, potentially affecting scleral stability and associating with non-syndromic early-onset high myopia [33]. In subject F#10, a novel LOXL3 variant (c.1580delC) was identified, accompanied by significant axial elongation, consistent with these findings. Subjects F#7 and F#10, carrying the P4HA2 c.1382C > G and LOXL3 c.1580delC variants, respectively, exhibited axial lengths (F#7: 25.3 mm/25.14 mm; F#10: 25.22 mm/25.19 mm) that exceed the age-matched average, supporting the pathogenic potential of these variants.

FBN1 encodes fibrillin-1, a protein associated with Marfan syndrome (MFS) and connective tissue elasticity, often accompanied by high myopia. Studies have suggested that variants in the C-terminal region of the FBN1 gene and TGF-β regulatory sequences are linked to increased axial length [34].In this patient (F#8), the axial length is significantly elongated (>26mm), yet typical systemic features of MFS (e.g., cardiovascular abnormalities) are absent, corroborating the findings of Flitcroft DI et al. [35]. Although the patient lacks systemic symptoms, systemic monitoring is required to confirm the delayed onset of Marfan syndrome (MFS). This novel variant may specifically promote axial elongation through scleral thinning, providing new evidence for the genetic heterogeneity of FBN1 in high myopia. Its pathogenicity warrants further investigation.

Interestingly, the P4HA2 gene is associated with autosomal dominant myopia 25 (MYP25) and encodes a critical component of prolyl 4-hydroxylase, an enzyme essential for collagen hydroxylation and scleral stability. Variants in this gene, such as c.419A > G (p.Q140R), have been linked to high myopia. The Human Phenotype Ontology (HPO) characterizes this condition as autosomal dominant inheritance, severe myopia, increased axial length, and an onset typically before 10 years of age.

Notably, in the F#5 family of this study, both the patient and the mother were heterozygous for the c.419A > G variant (Fig 3A) and exhibited mild to moderate myopia, in stark contrast to the severe phenotype (SE < −10.0D, AL > 26 mm) reported by Guo et al. (2015 [4]).This variability supports the perspective of Morgan et al. (2012) that myopia is regulated by both environmental and genetic factors [36].

The phenotypic variability, particularly with the P4HA2 c.419A > G variant, underscores the role of gene-environment interactions and incomplete penetrance. The phenotype is influenced by multiple factors, including genetic modifiers, environmental exposures (such as prolonged near work or reduced outdoor activity), and potential epigenetic interactions with other loci, indicating incomplete penetrance [36]. Future studies should investigate the interplay between genetic and environmental factors to achieve a more comprehensive understanding of the pathogenesis of myopia.

The novel variants identified in this study enrich the genetic landscape of high myopia. While variants in P4HA2 and FBN1 have been extensively documented, the roles of LOXL3, BBS2, and FZD4 in non-syndromic high myopia are less commonly reported. The young age of the cohort (median 9 years) and the variable expressivity of the implicated genes support the possibility that these patients may be in a pre-symptomatic stage. For syndromic genes (BBS2, FBN1, FZD4), systemic or retinal symptoms may manifest later, necessitating regular multidisciplinary evaluations. For non-syndromic genes (P4HA2, LOXL3), milder phenotypes may reflect early myopia progression, which could worsen with age or environmental triggers. Additionally, the small sample size (n = 56) and the lack of comprehensive phenotypic data and familial cosegregation analysis in some individuals may limit the in-depth evaluation of variant-specific phenotype associations. Future studies should validate the pathogenic potential of these variants in high myopia using larger cohorts and more complete familial data.

## Conclusion

This study identified five novel variants through exome sequencing and discovered two cases with phenotypes distinct from previous studies. These findings reveal the genetic heterogeneity of high myopia and provide new targets for genetic screening and intervention, which can be used to identify high-risk children and support the development of personalized preventive strategies, such as early optical interventions or lifestyle modifications. Future studies should validate the functional impact of these variants and integrate them into precision medicine for myopia.

## Supporting information

S1 Table Gene summary table. (XLSX)
